# Overexpression of p53 predicts colorectal neoplasia risk in patients with inflammatory bowel disease and mucosa changes indefinite for dysplasia

**DOI:** 10.1093/gastro/gov022

**Published:** 2015-06-10

**Authors:** Bela Horvath, Ganglei Liu, Xianrui Wu, Keith K Lai, Bo Shen, Xiuli Liu

**Affiliations:** ^1^Department of Anatomic Pathology, Cleveland Clinic, Cleveland, OH, USA,; ^2^Department of Geriatric Surgery, the Second Xiangya Hospital of Central South University, Changsha, China,; ^3^Department of Colorectal Surgery, Cleveland Clinic, Cleveland, OH, USA,; ^4^Department of Colorectal Surgery, the Sixth Affiliated Hospital of Sun Yat-sen University, Guangzhou, China,; ^5^Department of Pathology, University of Arkansas for Medical Sciences, Little Rock, AR, USA and; ^6^Department of Gastroenterology/Hepatology, Cleveland Clinic, Cleveland, OH, USA

**Keywords:** inflammatory bowel disease, ulcerative colitis, Crohn’s disease, colorectal neoplasia, p53, cytokeratin 7

## Abstract

**Background and aims:** We previously demonstrated a significant colorectal neoplasia risk in inflammatory bowel disease (IBD) patients with mucosal changes indefinite for dysplasia (IND) and the potential diagnostic utility of p53 and cytokeratin 7 immunohistochemistry in IBD-associated neoplasia. The primary aim of this exploratory study was to determine the predictive value of the two markers for neoplasia risk in the IBD-IND population.

**Methods:** We identified 44 eligible cases with IBD and IND in colon biopsy from our pathology database. We semi-quantified the expression of p53 and cytokeratin 7 in the colon biopsies by immunohistochemistry and correlated their expression, demographic information, and clinical features with colorectal neoplasia outcome.

**Results:** The mean age of the cohort was 46.6 ± 15.1 years, with 25 (56.8%) being male. The median follow-up was 101 months (range: 6–247) after IND diagnosis. Among these 44 patients, 11 (25%) progressed to neoplasia (low-grade dysplasia = 6; high-grade dysplasia = 2; cancer 3) at a median follow-up of 66 months (range: 19–145). Univariate analysis demonstrated that age and p53 overexpression were associated with progression to neoplasia.

**Conclusions:** Twenty-five percent of patients with IBD and IND developed colorectal dysplasia or cancer. Overexpression of p53 and age are associated with neoplastic progression.

## Introduction

It has been well established that patients with inflammatory bowel diseases (IBD), i.e. ulcerative colitis (UC) and Crohn’s disease (CD), face an increased risk of developing colorectal cancer (CRC) [1–4]. CRC causes death in 15% of IBD patients [5]. Current management methods have focused on the early detection of dysplasia (pre-cancerous change) in the colorectal epithelium as a marker for increased cancer risk and, thus, an indication for total proctocolectomy.

In the landmark paper by Riddell *et al.*, a standardized classification of dysplasia was proposed for the evaluation of biopsy specimens from patients with IBD [6]: the pathology report should clearly state the presence or absence of dysplasia. While most cases can be readily classified—either as negative or positive for dysplasia—the authors acknowledged a category of “mucosa indefinite for dysplasia (IND)” defined as “epithelial changes impossible to be classified as either unequivocally negative or positive for dysplasia”. While several studies reported that 4.9–33% of UC patients with low-grade dysplasia (LGD) progressed to advanced neoplasia [7–12], only one study included in its cohort a subgroup of IBD-IND patients in addition to IBD-LGD. Notably, this study observed that 28.6% of UC patients with IND progressed to high-grade dysplasia (HGD) or CRC in patients undergoing surveillance [8]. Additionally, the progression rate of LGD or IND has been reported as being dependent upon diagnostic accuracy, as misclassification of dysplasia in IBD patients significantly affects the rates of progression to advanced neoplasia [13]; however, specific diagnostic criteria for IND have not been clearly defined and this entity thus has the lowest rate of inter-observer agreement among grading pathologists—including gastrointestinal pathologists—as repeatedly reported in several studies [14–16], thus calling for risk-stratifying biomarkers in this diagnostically challenging IBD patient population.

We have previously shown that IBD patients with IND mucosal changes carried significant risk for progression to neoplasia: 25.2% of patients with IBD-IND developed dysplasia or carcinoma during a mean follow-up of 98.6 months, with an incidence of all neoplasia at 3.2 cases/100 person-years and an incidence of advanced neoplasia at 1.5 cases/100 person-years [14]. We also reported the diagnostic utility of p53 and cytokeratin 7 in IBD-associated neoplasia [17]. This study aimed to determine the predictive value of the two markers for neoplasia progression risk in an IBD-IND population.

## Materials and Methods

### Study population

The pathology database at the Department of Anatomic Pathology at Cleveland Clinic was searched for IBD, UC, CD, colitis, and IND for the years from 1990 to 2003. The decision to include cases only up to 2003 in this study was made because of the need for adequate follow-up. Patients who had dysplasia that (i) had been diagnosed previously, or synchronously at the time of IND diagnosis, (ii) had incomplete or missing medical records, (iii) were lost to follow-up (i.e. never returned to have clinical visits or hospital admission after the diagnosis of IND), or (iv) had been followed up for less than 6 months were excluded. Patients who had had a colectomy within 6 months after their IBD-IND diagnosis were also excluded. In addition, cases were excluded for which either (i) slides for histological review, or (ii) enough tissue for immunohistochemistry, were not available.

### Demographic and clinical variables

By careful chart review, data were collected on demographic and clinical variables, including age, gender, IBD subtype, duration of disease, extent of disease, and the presence or absence of primary sclerosing cholangitis (PSC).

### Histological interpretation and pathology review

The initial interpretation of IND was used in this study for the analysis of neoplasia progression, mainly to reflect the real-time status of patients as understood by their gastroenterologists as part of routine practice. Histology slides containing incident neoplasia in the follow-up colon biopsies were reviewed by the same gastrointestinal (GI) pathologist (X.L.).

### Immunohistochemistry for p53 and cytokeratin 7

Immunohistochemical staining for p53 and cytokeratin 7 was performed on whole tissue section from Hollande's fixed or formalin-fixed and paraffin-embedded tissue. Briefly, de-paraffinized tissue sections were stained with antibodies against p53 (clone DO-7, at 1:20 working dilution; Dako Corp., Carpinteria, CA) and cytokeratin 7 (clone OV-TL 12/30, diluted 1:40; Dako Corp., Carpinteria, CA), diaminobenzidine served as the chromogen. Positive and negative controls for these antibodies were included for the study.

**Figure 1.**
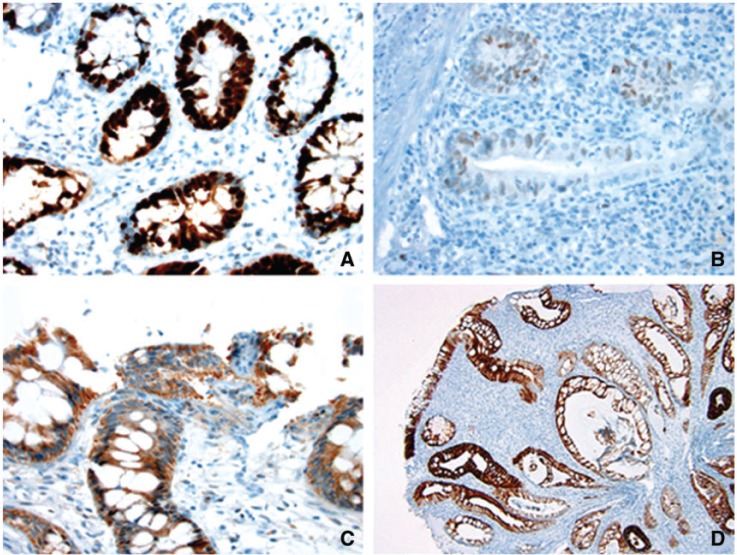
Representative images of strong nuclear p53 staining (A: immunoperoxidase stain × 400), weak nuclear p53 staining (B: immunoperoxidase stain × 400), strong cytoplasmic staining (C: immunoperoxidase stain × 400), and cytokeratin 7 expression (D: immunoperoxidase stain × 100) in colonic epithelium from patients with inflammatory bowel disease.

All immunohistochemical stains were evaluated by the same pathologist (X.L.), who was blinded to the neoplasia outcome. The presence or absence of nuclear staining was evaluated in the colon tissue present on the slides for p53. The expression of p53 was determined as a percentage of epithelial cells within a high-power field showing strong nuclear staining (Figure 1A), weak nuclear staining (Figure 1B), or strong cytoplasmic staining (Figure 1C). A composite p53 score for each case was obtained by the sum of (i) the percentage of epithelial cells with weak nuclear staining, (ii) three times the percentage of epithelial cells with strong nuclear staining, and (iii) the percentage of epithelial cells with strong cytoplasmic staining. Cytokeratin 7 expression was determined as the percentage of cells with membranous/cytoplasmic staining (Figure 1D).

### Outcome measurement

The primary endpoints of this study were incident neoplasia, which was defined as the presence of LGD, HGD, or CRC in the subsequent surveillance colonoscopy biopsy or at colectomy more than 6 months after diagnosis.

### Statistical analysis

Patients with an index diagnosis of IND, who were followed by surveillance colonoscopy, were included in this study. Time to progression was measured in months. Patients who did not develop LGD, HGD or CRC during follow-up were censored at the time of their last colonoscopy or colectomy. Descriptive statistics were computed for all variables; these included means and standard deviations (SD) or medians and interquartile ranges (IQR) for continuous factors, and frequencies for categorical factors. Comparisons of the distributions of the two groups' patients characteristics were made by using the two-tailed *t*-test or Wilcoxon rank sum test, as appropriate, for continuous variables and, for categorical variables, the chi-squared test or the Fisher exact test, as appropriate. The selection of cut-off points for p53 strong nuclear stain, p53 strong cytoplasm stain and composite p53 score, based on the risk for IBD-IND progression, was performed using the X-tile program (version 3.6.1, Yale University School of Medicine, New Haven, CT, USA) [18]. Probability of progression was estimated using the Kaplan–Meier method and compared using the log-rank test. Univariate analysis was performed using Cox proportional hazards regression. Unless specified, statistical analysis was performed using SPSS software version 16 (SPSS, Chicago, IL, USA). A *P*-value of less than 0.05 was considered statistically significant.

## Results

**Figure 2.**
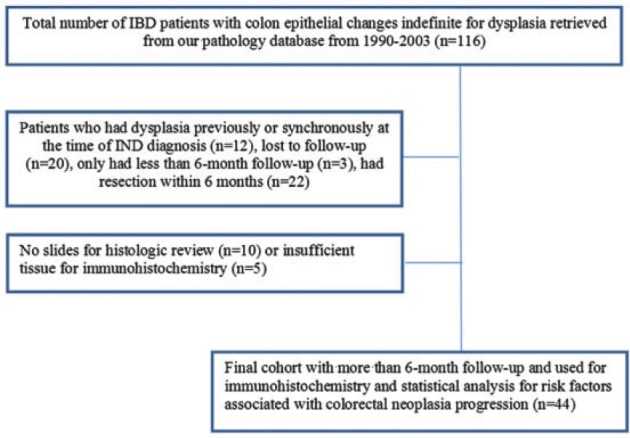
Selection process for study patients with inflammatory bowel disease and colonic epithelial changes indefinite for dysplasia.

A total of 116 IBD patients were identified with a diagnosis of IND on biopsy. Thirty-five patients were excluded because of a previous diagnosis of neoplasia (*n = *12), being lost to follow-up (*n = *20), or having follow-up shorter than 6 months (*n = *3). (See Table 1). for the flowchart of study IBD patients with a diagnosis of IND on colon biopsy.) Twenty-two patients who had colectomy within 6 months following the diagnosis of IND were also excluded. After excluding 10 further cases which lacked slides for histological review and 5 cases with insufficient tissue for immunohistochemistry, 44 patients with an index diagnosis of IND remained in this study. The selection process for the study cohort is shown in Figure 2. The demographic and clinical features of these patients are shown in Table 1.

**Table 1.** Demographics and clinical features of inflammatory bowel disease patients with mucosal changes indefinite for dysplasia

Characteristic	All cases(*n = *44)	IND without progression (*n = *33)	IND with progression (*n = *11)	*P*-value
Age, yrs	46.6 ± 15.1	43.4 ± 14.0	56.2 ± 14.7	0.031
Male, *n* (%)	25 (56.8)	17 (51.5)	8 (72.7)	0.3
IBD type, *n* (%)				0.31
Ulcerative colitis	38 (86.4)	27 (81.8)	11 (100)	
Crohn’s disease or indeterminate colitis	6 (13.6)	6 (18.2)	0 (0)	
Extent colitis (pancolitis), *n* (%)	21 (47.7)	15 (45.5)	6 (54.5)	0.6
Primary sclerosing cholangitis, *n* (%)	8 (18.2)	5 (15.2%)	3 (27.3%)	0.39
No. of interval colonoscopy (IQR)	4.0 (1.3–7.0)	3.0 (1.0–7.0)	7.0 (2.0–10.0)	0.23
No. of total follow-up biopsies (IQR)	12.0 (7.0–29.0)	12.0 (5.5–22.0)	29.5 (9.8–41.5)	0.06
Duration of colitis, yrs (IQR)	7.5 (2.0–12.8)	8.0 (2.5–14.0)	4.0 (0–10.0)	0.3
p53 weak nuclear stain, % (IQR)	10.0 (3.0–27.5)	10.0 (1.5–20.0)	20.0 (3.0–35.0)	0.17
p53 strong nuclear stain, % (IQR)	1.0 (0–5.0)	1.0 (0–3.0)	3.0 (0–10.0)	0.13
p53 strong cytoplasm, % (IQR)	0 (0–8.8)	0 (0–7.5)	2.0 (0–20.0)	0.34
Composite p53 score^a^ (IQR)	20.0 (5.8–44.0)	18.0 (5.0–36.0)	35.0 (17.0–85.0)	0.1
Cytokeratin 7, % (IQR)	3.5 (0–27.5)	2.0 (0–35.0)	8.0 (0–20.0)	0.81
Time to progression or last follow-up, months (IQR)	95.2 (42.4–129.2)	100.5 (54.4–134.1)	66.1 (24.7–86.1)	0.11
Time to last follow-up, months (IQR)	100.8 (62.7–135.7)	100.5 (54.4–134.1)	122.9 (66.1–145.0)	0.44

^a^A composite p53 score for each case was obtained by the sum of (i) the percentage of cells with weak nuclear staining, (ii) three times the percentage of cells with strong nuclear staining, and (iii) the percentage of cells with strong cytoplasmic staining.

CD = Crohn’s disease; IBD = inflammatory bowel disease; IC = indeterminate colitis; IND = indefinite for dysplasia; UC = ulcerative colitis

**Figure 3.**
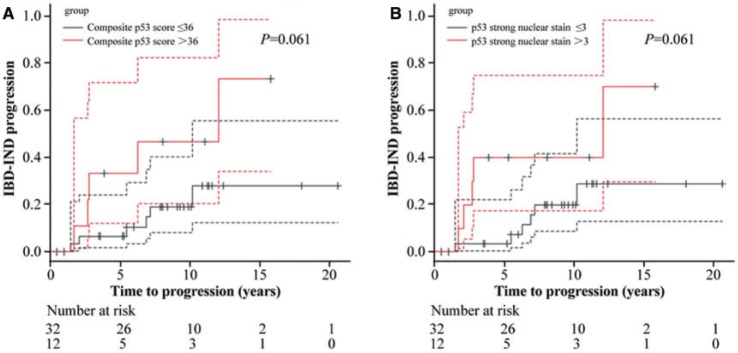
Overexpression of p53, defined either by composite p53 score (A) or percentage of epithelial cells with strong nuclear immunoreactivity (B) in colonic epithelium with indefinite for dysplasia as a risk factor for neoplasia progression in patients with inflammatory bowel disease by Kaplan-Meier curve.

The selected IBD patients were followed up in our hospital, using surveillance colonoscopy, for a median duration of 101 months (range: 6–247) and 93% of them had follow-up of at least 1 year. The median surveillance interval was 18 months (range: 6–139). Among these 44 patients, 11 (25%) progressed to neoplasia (LGD = 6; HGD = 2; cancer = 3) at a median follow-up of 66 months (range: 19–145). Univariate analysis confirmed that age was associated with neoplasia progression [hazard ratio (HR) = 1.07 for 1-year increase in age; 95% confidence interval (CI): 1.02–1.12; *P = *0.003] (Table 2). In addition, strong p53 nuclear staining (HR = 1.05; 95% CI: 1.01–1.09; *P = *0.009), strong p53 cytoplasmic staining (HR = 1.04; 95% CI: 1.01–1.08; *P = *0.025), and composite p53 score (HR = 1.01; 95% CI: 1.003–1.02; *P = *0.007) were associated with neoplasia progression (Table 2 and Figure 3). In contrast, weak nuclear staining of p53 and cytokeratin 7 expression were not associated with neoplasia progression (HR = 1.02; 95% CI: 0.99–1.04; *P = *0.19 for p53; HR = 0.99; 95% CI: 0.97–1.01; *P = *0.39 for cytokeratin 7) (Table 2). Other clinical characteristics and demographics—such as gender, extent of colitis, duration of colitis, or PSC—were not associated with progression of neoplasia in IBD-IND population (Table 2).

**Table 2.** Univariate analysis of risk factors associated with IBD-IND progression

Characteristics	Hazard ratio	95% confidence interval	*P*-value
Age, per 1-yr increase	1.07	1.02–1.12	0.003
Gender (male *vs.* female)	1.85	0.49–6.97	0.37
IBD type (IC or CD *vs.* UC)	N/A	N/A	N/A
Extent colitis (pancolitis *vs.* non-pancolitis)	1.37	0.41–4.56	0.61
Duration of colitis, per 1-yr increase	0.93	0.84–1.04	0.2
Primary sclerosing cholangitis (present *vs.* absent)	2.18	0.57–8.28	0.25
p53 weak nuclear stain, per 1% increase	1.02	0.99–1.04	0.19
p53 strong nuclear stain, per 1% increase	1.05	1.01–1.09	0.009
p53 strong cytoplasm, per 1% increase	1.04	1.01–1.08	0.025
Composite p53 score, per 1 increase	1.01	1.003–1.02	0.007
Cytokeratin 7, per 1% increase	0.99	0.97–1.01	0.39

CD = Crohn’s disease; IBD = inflammatory bowel disease; IC = indeterminate colitis; IND = indefinite for dysplasia; UC = ulcerative colitis; N/A = not applicable

## Discussion

It is well established that IBD carries an increased risk for developing CRC [1–4]. Detection of colorectal dysplasia by surveillance colonoscopy with extensive biopsy is the current standard of care for minimizing mortality from colon cancer in IBD patients; however, difficulties with diagnosis and uncertainty remain and may lead to a diagnosis of IND in some cases. Our previous study reported a risk of incident neoplasia in IBD patients with IND [10], in which about 25.2% of IBD-IND patients progressed to dysplasia or carcinoma during a median follow-up period of 101 months (range: 6–247).

We performed immunohistochemistry for p53 and cytokeratin 7 in 44 cases of colon biopsies with IBD-IND and investigated the value of these two markers in predicting neoplastic progression in IBD-IND because our previous study showed overexpression of p53 and cytokeratin 7 in IBD-associated neoplasia in a partially overlapping and partially complementary fashion [17]. In our current cohort of IBD patients with IND, 27% of cases had ≥ 5% epithelial nuclei with intense p53 expression; this rate is comparable with the results of a previous study that found 21% of colon biopsies with IND showed intense nuclear p53 immunoreactivity in ≥ 5% epithelial nuclei [19]. Our present study showed, by univariate analysis, that strong nuclear expression of p53 was associated with neoplasia progression. In addition we found, by univariate analysis, that strong cytoplasmic expression and a composite p53 score were associated with neoplasia progression. Our results indicate that the overexpression of p53—as analysed by Immunohistochemistry—predicts colorectal neoplasia progression in IBD patients with IND; this finding is consistent with previous studies that found p53 overexpression (defined as intense nuclear immunoreactivity) was a risk factor for colorectal neoplasia progression in UC and CD [20–22]. Although a recently published paper suggested that p53 nuclear staining and alpha-methylacyl-CoA racemase (AMCAR) immunoreactivity in biopsies with flat LGD and IND predicts progression to advanced IBD-associated neoplasia in a time period of 19 months [22], our study is the first to demonstrate the predictive value of p53 overexpression by immunohistochemistry in a specific, histologically challenging population, i.e. IBD patients with IND. Further, our results of a composite p53 score suggest a possibility of quantitative measurement of p53 proteins in colon biopsies by other means, such as enzyme-linked immunosorbent assay (ELISA), to provide more objective measurement.

Although our previous study showed overexpression of cytokeratin 7 in IBD-associated neoplasia [17], in this study cytokeratin 7 expression was not associated with neoplastic progression in the IBD-IND cohort. This result is not a surprise, as cytokeratin 7 overexpression may occur in reactive and neoplastic colonic epithelium in IBD [17, 23].

Neoplastic progression was not associated with patient gender, IBD subtype, extent of colitis, duration of disease, presence of PSC, number of colonoscopy procedures, or number of biopsies, similarly to findings reported in a previous study of LGD and IND [8]. In our previously studied IBD-IND cohort [14], we identified age as a risk factor for neoplastic progression. This association is also seen in our current subgroup of IBD-IND analysed for p53 and cytokeratin 7, suggesting that the current subgroup is indeed representative of the entire IBD-IND cohort in our previous study. Notably, the number of biopsies for those with IND who progressed was greater than for those who did not progress (*P = *0.06), suggesting a potential confounding effect. However, because only 11 patients progressed in our cohort, a multivariate analysis could not be performed to address such an effect.

There are limitations to our study. Our data are limited by the availability and quality of the clinical information found within the medical records; for example, many cases lacked detailed endoscopic reports, preventing us from determining whether the IND biopsy was from a lesion or from flat mucosa, and from correlating the location of index IND with the location of incident neoplasia. However, this may not significantly compromise our conclusions, as p53 mutation—detected either by genotypic mutation assay or Immunohistochemistry—has been shown to occur in non-cancerous tissue from UC [19, 24], and has previously been associated with colorectal neoplasia progression in UC and CD, regardless of the biopsy site [20, 21]. In addition, many cases lacked a detailed history, such as a family history of colorectal cancer and medication history. The rate of incident neoplasia in our study may represent an overestimation because of the referral bias, and we could not completely rule out a third-party diagnosis of dysplasia prior to the IND diagnosis at our institution. Similarly, the study population was based on a highly specialized tertiary care center and conclusions from this study may not be applicable to other practice settings. In addition, the number of cases we used for immunohistochemistry and the number of cases with progression were small, precluding multivariate analysis. Further the determination of p53 and cytokeratin 7 was only semi-quantitative.

In summary, our study reaffirms the value of IND as a diagnostic category for IBD colon biopsies. The diagnosis of IND carries a significant incidence rate for neoplasia in IBD patients. IBD patients with IND should be followed-up by repeat colonoscopy after a short interval (within months) and then managed according to the findings of the repeat biopsy. In addition to patient age, immunohistochemical stain for p53, performed on the colon biopsies with IND, may be helpful in further identifying patients who have higher risk of progression. Large-scale studies, with multivariate analysis, are needed to confirm our findings.
